# Chronic respiratory disease mortality and its associated factors in selected Asian countries: evidence from panel error correction model

**DOI:** 10.1186/s12889-020-10042-7

**Published:** 2021-01-06

**Authors:** Emerson Augusto Baptista, Sudeshna Dey, Soumya Pal

**Affiliations:** 1 Asian Demographic Research Institute (ADRI), Shanghai University, Shanghai, 200444 China; 2 Karnataka Health Promotion Trust (KHPT), Bengaluru, Karnataka 560044 India; 3 Indian Institute of Management Bangalore (IIMB), Bengaluru, Karnataka 560076 India

**Keywords:** Mortality, Chronic respiratory diseases, Associated factors, Asian countries, Global burden of disease study 2017, Panel error correction model

## Abstract

**Background:**

Chronic Respiratory Diseases (CRDs) in Asian countries are a growing concern in terms of morbidity and mortality. However, a systematic understanding of the increasing age-adjusted mortality rate of chronic respiratory disease (CRD) and its associated factors is not readily available for many Asian countries. We aimed to determine country-level factors affecting CRD mortality using a panel error correction model.

**Methods:**

Based on data from the Global Burden of Disease Study 2017, we estimated the trends and distribution of CRD mortality for selected Asian countries from 2010 to 2017. Furthermore, we evaluated the relationship between CRD mortality and Gross Domestic Product (GDP) per capita, average years of schooling, urbanization, and pollutant emission (PM2.5 concentration) using a fixed-effect model. We corrected the estimates for heteroscedasticity and autocorrelation through Prais-Winsten adjustment along with robust standard error.

**Results:**

Between 2010 and 2017, approximately 21.4 million people died from chronic respiratory diseases in the countries studied. Age-standardized crude mortality rate from CRDs in the period had minimum and maximum values of 8.19 (Singapore in 2016) and 155.42 (North Korea in 2010) per 100,000 population, respectively. The coefficients corrected for autocorrelation and heteroskedasticity based on the final model of our study (Prais-Winsten), showed that all explanatory variables were statistically significant (*p* < 0.001). The model shows that the 1% increase in GDP per capita results in a 20% increase (0.203) in the CRD mortality rate and that a higher concentration of air pollution is also positively associated with the CRD deaths (0.00869). However, an extra year of schooling reduces the mortality rate by 4.79% (− 0.0479). Further, rate of urbanization is negatively associated with the CRD death rate (− 0.0252).

**Conclusions:**

Our results indicate that both socioeconomic and environmental factors impact CRD mortality rates. Mortality due to CRD increases with rising GDP per capita and decreases with the percentage of the total population residing in urban areas. Further, mortality increases with greater exposure to PM2.5. Also, higher years of schooling mitigate rising CRD mortality rates, showing that education can act as a safety net against CRD mortality. These results are an outcome of sequential adjustments in the final model specification to correct for heteroscedasticity and autocorrelation.

## Background

The growth of chronic respiratory diseases (CRDs) in recent decades has changed morbidity and mortality trends and has become a problem worldwide [1]. CRDs represent a group of diseases characterised by abnormal conditions of the respiratory system, including chronic obstructive pulmonary disease (COPD) and asthma, the most common, as well as pneumoconiosis, interstitial lung disease, and pulmonary sarcoidosis, which affect people of all ages. Among the main risk factors identified for CRDs are tobacco smoking, air pollution (indoor and outdoor), allergens, and occupational risks and vulnerability [2].

In the last twenty-seven years of available data, although the percentage has remained stable at around 7% in relation to all causes of death, the estimated number of people who died annually from CRDs jumped from 3,317,205 in 1990 to 3,914,196 in 2017, an increase of ~ 18%. The majority of CRD deaths in this period occurred on the Asian continent, which in 2017 recorded approximately 75% of all cases, a slightly lower percentage than in 1990 (~ 77%) [3]. Therefore, and considering that Asia represents approximately 60% of the world’s population, CRDs constitute a serious public health problem that strongly affects countries in the region [4].

In recent years, Asia has seen a booming economy, an unprecedented amount of rural to urban migration, and rapidly growing cities accompanied by increasing exposure to environmental pollutants. In addition, other structural issues have created a huge threat to the ecological environment for healthy living, which contributes to a rising burden of disease, particularly CRDs, and, consequently, leads to huge economic loss [5–7].

To identify interventions, plan priorities, and strategically allocate resources to reduce the burden of disease, it is essential to understand the factors that drive CRD-related mortality. In Asia, as well as elsewhere, studies of CRD-related mortality are limited either by data or mainly confined to individual countries. Moreover, studies are focused on a specific cause, such as COPD, asthma or tuberculosis [8–15]. According to Soriano et al. [16], there is a lack of studies summarizing the burden of disease or mortality of all CRDs worldwide over an extended period. Added to this, there are still known and unknown aspects related to risk factors. While smoking (including second-hand smoke), air pollution (indoor and outdoor), allergens, and occupational risks are well-known and widely studied risk factors for chronic respiratory diseases [13, 16], socioeconomic factors, such as gross domestic product (GDP) per capita and schooling, still have a literature gap capable of contributing and advancing the understanding of CRDs. Finally, there is a paucity of evidence regarding the various levels of factors that affect CRD mortality rates, as well as cross-country studies that fail to acknowledge the challenges of statistical modeling, such as serial autocorrelation and heteroskedasticity. However, measurement outcomes from broad disease categories reflect the responsiveness of healthcare system in a country and serve as an important starting point [17].

With this in mind, the goal of this study is twofold. Substantively, we aim to to evaluate the relationship between CRD mortality rates and some possible predictor variables across Asian countries. Methodologically, we to try to develop the rationale for the model selection in cross-country panel data, test the plausible violations of assumptions for regression, and correct the estimates of coefficients for the same. The estimates obtained were corrected for non-compliance of assumptions in linear models to answer the following research questions: (1) What are the pattern, dynamics and trends of CRDs mortality in the period? (2) Is there an association between age-standardized crude mortality rate from chronic respiratory diseases and any of the explanatory variables studied? (3) What are the possible limitations in statistical models used in cross-country panel data and what are the ways to address them?

## Methods

### Data and measures

The country-level age-standardized crude mortality rate from chronic respiratory diseases (CMRCRD) is the dependent variable of this study and can be calculated as follows:

1 $$ CMRCRD=\sum {m}_i^j{C}_i^s $$

where ${m}_i^j$ is the age-specific mortality rate from chronic respiratory diseases in each age group *i* and country *j*, and ${C}_i^s$ is the proportion of the population in each age group *i* of the standard population, which, in this study, is the world population in 2010. Data, which is cause-specific as well as age-specific (in 5-year age groups up to 95 years or more), were obtained from the Global Burden of Disease (GBD) Study 2017 [3], coordinated by the Institute for Health Metrics and Evaluation (IHME) and publicly available online.

The GBD study was created to provide comprehensive and comparable global health metrics. Estimates of cause-specific mortality, burden of diseases, injuries, and risk factors are reported by year (1990–2017), location (195 countries and territories, and at the subnational level for a subset of countries), age, and sex. The GBD study also supplied the population estimates used in this paper. Data sources used to produce these estimates came from 1257 census and 761 population registry location-years.

The explanatory variables of the study are gross domestic product (GDP) per capita, schooling, urbanization, and air pollution.

We obtained the gross domestic product per capita (GDP per capita) and the expected years of schooling from the United Nations Development Programme [18]. The first is measured in purchasing power parity (2011 PPP $) as result of GDP divided by the total population in the same year. This is one of the most widely used socioeconomic predictors of mortality / health, and this relationship has been widely discussed in the literature [19–27]. The second refers to the number of years of schooling that a child of school entrance age (under age 7) can expect to receive if prevailing patterns of age-specific enrolment rates persist throughout the child’s life. A vast literature has persistently shown the inverse association between educational attainment and mortality / morbidity, almost invariably indicating that individuals with better education are healthier and live longer [25, 28–33].

Annual percentage of population at mid-year residing in urban areas was obtained from the United Nations Department of Economic and Social Affairs [34]. Urbanization is an important factor in mortality and health, as it is associated with a more sedentary lifestyle, a diet richer in salt, sugar, and fat, and tobacco addiction. Added to this is the problem of air pollution, one of the main problems observed in urban areas, with adverse health implications resulting in particular from the combustion of fossil fuels from vehicular traffic [25, 35–38]. However, higher urbanization leads to better access to modern health facilities owing to concentration of facilities in urban areas [39].

Lastly, population-weighted exposure to ambient PM2.5 pollution comes from the State of Global Air report [40]. This variable is defined as the average level of exposure of a country’s population (in both urban and rural areas) to mean annual concentrations of suspended particles measuring less than 2.5 μm in aerodynamic diameter (μg/m^3^). These particles are capable of penetrating deep into the respiratory tract and causing severe health damage. It has been well established in the literature that air pollution is one of the most important risk factors for chronic respiratory disease [41–46].

### Level of analysis

The analysis in the study is based on information from 29 Asian countries, including Iran (Western Asia); Afghanistan, Kazakhstan, Kyrgyzstan, Tajikistan, Turkmenistan, and Uzbekistan (Central Asia); Bangladesh, Bhutan, India, Nepal, and Pakistan (Southern Asia); China, Japan, Mongolia, North Korea, and South Korea (Eastern Asia); and Brunei, Cambodia, Indonesia, Laos, Malaysia, Maldives, Myanmar, Philippines, Singapore, Sri Lanka, Thailand, and Vietnam (Southeastern Asia). That these countries cover and represent huge socioeconomic, demographic, geographic, environmental, and epidemiological diversity is well known. However, Asia is a region where CRD mortality may be especially problematic due to the stages of growth and development that many countries have experienced, particularly from the second half of the twentieth century.

For purposes of analysis, we use a time series from 2010 to 2017. Deaths from chronic respiratory diseases and population were organized by age (in 5-year age groups up to 95 years or more). We then calculate age-standardized death rates per 100,000 for each country using the world population in 2010 as the standard.

### Methodology

This section describes the econometric framework used to establish the relationship between chronic respiratory diseases mortality rates and set of covariates described earlier. We used the log-linear functional form to estimate all regression equations. Equation 1 provides standard formulation of the model:

2 $$ {y}_{ct}=\theta {z}_{ct}+\upbeta X{\prime}_{ct}+{\sigma}_c+{\varepsilon}_{ct} $$

where *y*_*ct*_ is log of age-standardized crude mortality rate from chronic respiratory diseases (CMRCRD), *z*_*ct*_ is log of the explanatory variable (GDP per capita or average years of schooling or rate of urbanization or concentration of particulate matters), and *X*′_*ct*_ comprises the other covariates as the linear function for country *c* and time *t*. *σ*_*c*_ denotes the country effect, where its randomness is tested to identify the true model. The logarithmic transformation of CRD mortality rates and GDP per capita is based on the assessment of SD within and between. Due to the inconsistent scale of variation of the variables, it was necessary to make the log transformation of high-variance variables. Therefore, to align the scale of variation in the model, we logarithmically transformed the previously mentioned variables. Apart from the mathematical transformation, we provide the rationale for model selection below.

Based on the nature of *σ*_*c*_, three prominent models used in the panel data setup were tested: pooled, fixed, and random effect models [47]. Model selection was based on three criteria: goodness of fit, significant difference between countries, and exogeneity of the cofactors.

In order to measure the goodness of fit, Akaike’s Information Criteria (AIC) were used, which required the utilisation of the maximum likelihood (ML) method to estimate the coefficients [48]. Apart from the goodness of fit, we tested appropriateness amongst the pooled and random effect models, based on the share of sum of squares across countries out of the total sum of squares. The Breusch-Pagan Multiplier Test suggested that the variation between countries explains a significantly larger portion of total variation [49]. This implied that the random effect model is more suitable than the pooled model. Further, exogeneity of cofactors plays an important role to estimate the consistent and unbiased coefficients. In Eq. (1), *σ*_*c*_ controls for the effect due to countries, which makes the other coefficients independent of the error term. However, estimating the parameters for this will result in loss of degrees of freedom, which necessitates elimination. Elimination of *σ*_*c*_ can be performed using either the partitioned regression or putting it in the error term. The selection of either model, also known as the fixed effect and random effect regressions, respectively, was conducted through the Hausman Test [47]. The test suggested that the correlation between regressor and error term is significant, that is, resulting in endogeneity, and that, therefore, the fixed effect model is the most appropriate to estimate the relationship between CRD mortality rates and the set of independent variables.

The panel setup may present challenges of heteroskedasticity and autocorrelation. Non-constant variance may result from the inherent characteristics of the study subjects [50]. The outcome variable in the model is a measure of population health, that is, it is a function of overall health infrastructure, which is not explained in the model. This presents a situation where the variance of error may depend on the structural health conditions of a country and, therefore, can vary widely across them [51]. Consequently, the Modified Wald Test is conducted to test for heteroskedasticity. The test result suggests that the existing fixed effect model is violating the assumption of homoskedasticity. Therefore, due to the presence of heteroskedasticity, we resort to robust standard errors to remove the bias. In addition, serial autocorrelation was also tested as to its violation of the regression assumptions, since its presence can result in an inconsistent estimate for the coefficients. We did this through the unit root test, which suggested the presence of serial autocorrelation. This requires adjusting the covariance matrix in order to get rid of the autocorrelation. Thus, we use the Feasible Generalised Least Square (FGLS) Model to estimate the correlates, but the model fails to consider the heteroskedasticity issue present in the panel setup. Hence, to estimate the coefficients adjusted for both autocorrelation and heteroskedasticity, we resort to a model proposed by Prais-Winsten [52]. Prais-Winsten estimation has proven to be an effective method in the presence of autocorrelation and heteroskedasticity [53]. The estimation corrects for autocorrelation and allows for robust standard error which adjusts the heteroskedasticity in the panel data [54]. In other words, the estimates of the coefficients are consistent and lead us to believe that a fixed effect model with error correction is the most suitable model for our study.

## Results

Table 1 presents the mean, standard deviation, minimum, and maximum of the variables used in the study. The observed mean of deaths from CRDs is 53.75 per 100,000 population. The summary statistics of the independent variables indicate that the mean of GDP per capita, years of schooling, urbanization and air pollution are $15,535.66, 12.63 years, 48.14%, and 36.55 μg/m^3^, respectively. The assessment of second-order moment (standard deviation) is performed to analyze the variation in dependent and independent variables across time and countries. The standard deviation (SD) within denotes the variation across time (temporal variation), whereas the SD between denotes the spatial variation. Higher share of SD between, compared to SD within, establishes that the spatial variation dominates over the temporal variation. For instance, in the case of CRD mortality rates, the SD between is 38.87, while the SD within is 2.96. The dominance of spatial variation over the temporal variation is also established statistically by Breusch-Pagan Multiplier Test. Further, the descriptive analysis in Table 1 provided the basis for regression model in terms of mathematical function required for each variable in the model.

**Table 1:** Descriptive statistics, overall

Variable		Mean	Std. Dev.	Min	Max
Chronic respiratory diseases mortality rates	overall	53.75	38.40	8.19	155.42
	between		38.87	9.30	147.92
	within		2.96	44.78	64.80
GDP per capita	overall	15,535.66	19,629.60	1614.00	85,535.00
	between		19,888.48	1777.25	79,151.75
	within		1478.52	8499.91	21,918.91
Years of schooling (mean)	overall	12.63	1.96	7.50	16.90
	between		1.96	8.01	16.64
	within		0.33	11.24	13.62
Urbanization (%)	overall	48.14	21.86	16.77	100.00
	between		22.17	18.02	100.00
	within		1.08	43.74	52.47
PM2.5 Annual Population Weighted Concentration	overall	36.55	22.63	5.90	101.00
	between		22.79	6.70	98.25
	within		2.93	22.80	45.80

Note: Total Observations: 232, Total Countries: 29, Years: 8

In Table 2, we present a static description of the selected covariates by intensity of mortality due to CRD. However, regression analysis is required to show the effect of the rate of change in GDP over the rate of change in CRD-based mortality. The rate of change in GDP per capita is much higher in countries with high mortality compared to countries with low mortality. For example, India has an average growth rate of 5–6% y-o-y, while Japan experiences a growth in GDP per capita of 1–2%. However, when comparing CRD mortality rates, India experiences a much higher rate than Japan, even though India is witnessing higher growth rate. Therefore, the regression model is utilized to estimate the effect of temporal variation in the growth rate of GDP per capita on CRD mortality. Additionally, unlike descriptive analysis, multivariate analysis controls for the confounding effects and shows the adjusted effect (ceteris-paribus). The inclusion of variables in the model, such as urbanization rate and schooling, controls for the spatial variation.

**Table 2:** Mean of selected covariates across countries with High, Medium and Low CRD Mortality

Covariates	High Mortality	Medium Mortality	Low Mortality
GDP per capita($)	4665.81	16,433.1	24,265.35
Urban Population (in %)	34.01	43.06	67.08
Schooling Years	10.98	13.06	13.83
PM2.5 (μg/m3)	58.13	23.23	28.38

**Fig. 1:**
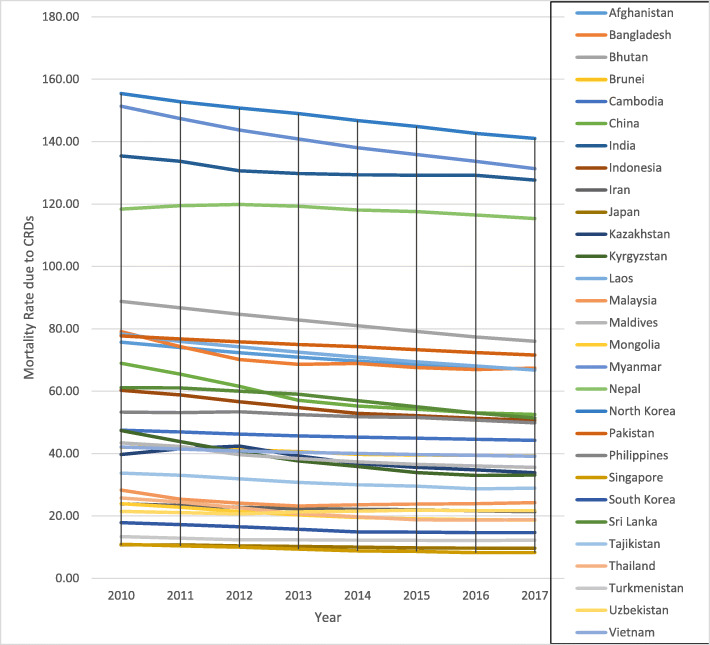
Temporal Variation in CRD mortality rates

Figure 1 shows the pattern, dynamics and trends of CRD mortality rates for each country in the study period. High mortality countries, such as North Korea, Myanmar, India, and Nepal, show stagnancy or a decline. The same pattern is seen for countries with low mortality rates, such as Japan, Singapore, South Korea, and Turkmenistan.

Table 3 compares the results and goodness of fit obtained from pooled ML, fixed effect and random effect models. Based on the AIC values and Hausman Test, we selected the fixed-effect model. The coefficient values for the fixed-effect model suggest that with an increase in the PM2.5 concentration, the mortality rate increases, whereas increases in GDP per capita, urban population and schooling years reduce the mortality rate. However, the sign and magnitude of the coefficients presented are inconsistent due to the presence of autocorrelation and heteroskedasticity.

**Table 3:** Testing the Model

Log (GMR)	(1) Pooled OLS	(2) Fixed Effect	(3) Random Effect
Log (GDP per capita)	0.332***	− 0.191***	− 0.161***
	(0.0670)	(0.0506)	(0.0495)
Years of schooling (mean)	−0.0648***	−0.0426***	− 0.0407***
	(0.0221)	(0.0128)	(0.0120)
Urbanization (%)	−0.0276***	−0.00915**	− 0.0116***
	(0.00268)	(0.00435)	(0.00346)
PM2.5 (concentration)	0.0121***	0.00346**	0.00395***
	(0.00156)	(0.00149)	(0.00140)
Constant	2.349***	6.266***	6.067***
	(0.512)	(0.428)	(0.417)
Observations	224	224	224
AIC	255.47	− 784.44	− 558.58
Number of Countries^a^		28	28

Note: *** *p* < 0.01, ** *p* < 0.05, * *p* < 0.1

^a^GDP value not available for North Korea

Table 4 presents the coefficients corrected for autocorrelation and heteroskedasticity based on the final model. The coefficients reported for the Prais-Winsten model show that the logarithmic mortality rate due to CRDs increases by 0.203 with the increase in log GDP per capita. Similarly, the marginal effect of PM-2.5 on the log mortality rate is estimated as 0.00869. However, the increase in schooling and urbanization has a negative association with the CRD mortality rates. For instance, the increase of one unit in the mean years of schooling reduces the log mortality rate by 0.0479, which presents itself as a resilience factor. To conclude, greater economic growth and exposure to PM-2.5 concentration in the atmosphere are increasing the fatality due to CRD. However, the negative effects can be reduced by literacy and urbanization.

**Table 4:** Estimates corrected for heteroscedasticity and autocorrelation

Log (GMR)	(1) Non-Robust SE	(2) Robust SE	(3) FGLS	(4) Prais-Winsten
Log (GDP per capita)	− 0.191***	− 0.191*	0.305***	0.203***
	(0.0506)	(0.0959)	(0.0204)	(0.0658)
Years of schooling (mean)	−0.0426***	−0.0426	− 0.0574***	− 0.0479***
	(0.0128)	(0.0267)	(0.00572)	(0.0185)
Urbanization (%)	−0.00915**	−0.00915	− 0.0270***	−0.0252***
	(0.00435)	(0.00968)	(0.000435)	(0.00218)
PM2.5 (concentration)	0.00346**	0.00346**	0.0117***	0.00869***
	(0.00149)	(0.00143)	(0.000315)	(0.00114)
Constant	6.266***	6.266***	2.493***	3.324***
	(0.428)	(0.680)	(0.135)	(0.480)
Observations	224	224	224	224
AIC	−784.44	− 786.44		
Number of Countries^a^	28	28	28	28

Note: *** *p* < 0.01, ** *p* < 0.05, * *p* < 0.1

^a^GDP value not available for North Korea

## Discussion

The significant number of deaths and morbidities due to CRDs in Asian countries warrant a comprehensive investigation of associated factors, from the individual level up to the country level. Researching this problem can make a significant contribution to the improvement of global public health. However, very few studies have focused on the assessment of risk factors associated with CRDs across multiple countries, and most of these lack a differential analysis of public health factors. This study explored the effect of GDP per capita, years of schooling, urbanization, and pollutant emission as socioeconomic and environmental factors on CRD-related mortality in 29 Asian countries, where dynamic growth has taken place at the cost of respiratory health. We further determined the rationale for the model-building to establish the relationship between CRD mortality rates and selected factors. The results highlight that both socioeconomic and environmental factors impact CRD mortality. This emphasises the role of reductions in air pollution, planned urbanization, and investment in human capital in the form of education to mitigate the risk of respiratory disease. These risk factors combine into a series of pathways that result in different types of CRDs, such as COPD, asthma, pneumoconiosis, and interstitial lung disease [55].

The major underlying factor for the rise in CRD mortality in Asian countries is rampant economic growth, which has resulted in a drastic changes in lifestyles. Thus, people in Asia became more exposed to fatal diseases such as CRD [6, 13, 14, 56]. Our results show that the annual increase in GDP per capita increases the CRD mortality rates. We speculate that the most likely cause is that GDP represents market consumption opportunity. Commonly, increasing GDP is associated with improved health facilities or increased consumption of calories and micronutrients, which are beneficial for the health outcomes of a country. On the other side, they can also lead to an unexpected increase in the number of diseases linked with prosperity. There may be an increased demand for goods associated with health risk, such as tobacco and alcohol, shifts in dietary structure and adoption of a more sedentary lifestyle [57]. Additionally, GDP growth also comes at the cost of environmental degradation [58–60]. Evidence suggests that higher growth in GDP has increased the level of pollutant emissions, which eventually leads to different types of CRDs and related deaths in many countries, particularly in Asian regions [55]. However, our results confirmed that the notion of GDP growth alongside the reduction in CRD deaths depends on the distribution of gains in the population. Also, the public health services and infrastructures of Asian countries might play a decisive role by comprising improved public health sectors as an integral part of comprehensive growth [57, 61]. This means that the relationship between mortality and GDP per capita growth is driven by the extent of public expenditure [61]. Controlling for the fixed effect of public health expenditure, which is in our case is the mean years of schooling, shows that the increase in GDP per capita increases CRD mortality.

Consistent with previous studies [13, 62–64], our results showed that PM2.5 is significantly positively correlated with CRD mortality, suggesting that higher exposure to particulate matter increases the mortality rate. In the last decade, many countries of Asia experienced increasing air pollution from both industrial and motor vehicle emissions [65]. The pollution also comes from burning of crop residues and bush fires in various Asian countries [66, 67]. Our findings suggest that there is a need for intervention to reduce air pollution in Asian countries and thereby limit premature mortality.

Very few studies have assessed the effect of urbanization on a range of CRDs. We found that the percentage of the total population residing in urban areas plays a key role as it reduced the mortality rate due to CRD. A higher percentage of urban dwellers is associated with a lower risk of mortality from CRD. Urbanization in Asian countries is accompanied by higher economic growth, favouring upward mobility in incomes [68]. This resulted in declining mortality as the quantum of shock from morbidity on a household budget was reduced with higher household savings and smoothing of consumption [69, 70]. It is observed that urban dwellers transition to cleaner fuel, which reduces the risk of CRD mortality [71]. Also, a gap exists in the transition rate between rural and urban areas, which provides an advantage to city dwellers. However, urbanization is associated with increased air pollution, which increases the risk of morbidity due to CRD but the associated mortality is reduced due to access to health care facilities and technologies [39].

Our results also suggest that a higher number of school years mitigates rising CRD mortality, showing that there is a negative association between them, and that education can act as a safety net against CRD mortality rates. A potentially important mechanism is that increased years of schooling is related to higher wages and earnings [72], which further enables individual to purchase better health care and creates awareness about the utility of health care [73, 74]. Further, from the structural lens, there is a possibility that higher investment in human capital reduces the risk of mortality by increasing employment opportunities and thereby broadening the safety net for individuals [75].

However, to obtain the best estimate for coefficients, we thoroughly discussed different model specifications. We observed inconsistency in the estimates across model specifications, which implies the need for appropriate rationales for model selection. Thus, error correction contributes significantly to the achievement of consistent and unbiased estimates.

Based on the nature of cross-country panel data, we expected that the error in the model might exhibit autocorrelation and heteroskedasticity. The expectation was based on the following two arguments: 1) The information was recorded for each year of the selected period (2010–2017). The observation in the current year has a significant likelihood of being related to the previous year’s value [76]. For illustration, the outcome variable, the mortality rates due to chronic respiratory disease, observed in time *t* can be related with the observation at *t* − 1 due to stickiness in the mortality rate for any specific region or country. Similar temporal autocorrelation has been observed in the past with respect to mortality for a given area [77]. 2) The argument for the presence of heteroskedasticity is suggested by the fact that countries can be ranked based on their mortality rates. This ranking allows for the clustering of countries, leading to heteroskedasticity. For example, countries like Japan, South Korea, and Singapore experience the lowest mortality rates, forming one group. In contrast, countries, such as India, Bangladesh, and Pakistan show a high mortality rate, and are hence placed in another group. The analysis of variance with the groups based on rankings suggest that variance within a group is significantly lower than it is across the groups. This results in heteroskedasticity of the error term in the model and therefore requires separate treatment.

However, the treatment of error to adjust for autocorrelation and heteroskedasticity is preceded by the selection of model. The selection of model was based on heterogeneity across the countries and endogeneity in the model. Based on the individual effect or heterogeneity of countries, the entire model can be treated as an ordinary linear model, whose parameters are estimated using maximum likelihood. We tested three plausible linear models: pooled regression, fixed effects and random effects. First, we tested whether the country fixed effects are constant terms. The Breusch-Pagan Multiplier Test suggests the fixed effect to be stochastic; therefore, the pooled regression model is discarded in favour of the random effect model. A subsequent challenge was to test the correlation between unobserved country effect and other covariates. In other words, we tested for endogeneity in the model. Identification of endogeneity is an essential step while developing the rationale for model selection, as it determines the form of unobserved country effects in the model. Further, inaccurate specification of country effects in the model results in inconsistent estimation of model coefficients [47]. Therefore, it was necessary to test the orthogonality of unobserved country-effects with the covariates. The Hausman test rejected the hypothesis of orthogonality, suggesting the fixed effect model to be ideal. Our finding is in alignment with the previous literature on cross-country studies which found non-orthogonality of unobserved country effects with the cofactors, such as GDP per capita, the concentration of air pollution, etc. [78, 79].

However, our analyses are also subject to limitations. The first, which is more general and has been reported elsewhere, concerns GBD studies and CRD estimates [80, 81]. Many scholars describe GBD estimates as a “black box”, which, from a scientific point of view, makes it impossible for anyone to replicate or verify the estimates. The second one is that this study does not incorporate other major risk factors associated with CRD mortality, such as tobacco smoking, air pollution (indoor), and allergens, although we argue that a good understanding of the selected variables is fundamental, as they are important predictors of mortality and health [19–21, 25–28, 32, 33, 35, 36, 41, 45, 46]. Finally, and although we have tried to eliminate all biases, it is important to note that even the most complex statistical models can have limitations.

## Conclusion

Our results indicate that both socioeconomic and environmental factors impact CRD mortality rates. The Prais-Winsten model shows that the mortality due to CRD increases with rising GDP per capita and decreases with the percentage of the total population residing in urban areas. These results may initially appear to be a counterintuitive. However, if we consider this from the perspective that many of the countries studied are middle-income countries, that is, are still in the process of development and, consequently, of an increase in GDP, this result may seem natural. At the same time, it is assumed that urbanization is associated with increased air pollution. However, the impact of pollution on the risk of morbidity due to CRD is greater that its impact on mortality, since urban areas are expected to offer better access to health care facilities and technologies. Nevertheless, CRD mortality rates show a positive association with PM2.5, which suggests that mortality increases with greater exposure to particulate matter. Additionally, greater schooling mitigates the rising CRD mortality rates, showing that they are negatively correlated and that education can act as a safety net against CRD mortality.

The results are an outcome of sequential adjustments in the final model specification to correct for heteroscedasticity and autocorrelation, since when evaluating the relationship between CRD mortality rates and some possible predictor variables across Asian countries, we observed the presence of these elements, which suggests the need for correction while modelling. Hence, we estimated the coefficients using a feasible generalised least squares model with robust standard error. The detailed discussion of the rationale for model selection highlighted the internal validity problems, which were corrected, making the coefficients of the final model trustworthy.

In addition, we argue that this study provides useful clues for policymakers establishing effective public health planning and measures for the prevention of deaths from chronic respiratory disease. The reduction of CRD mortality rates, for example, can increase life expectancy enables people to contribute to the economy of a country and its regions for longer periods. Improvements in air quality, which many Asian countries are making progressively and in a remarkable manner, can make a substantial contribution to reducing deaths from CRD. Overall, this paper presents important findings that countries can benefit from in the short, medium, and long term.

## Recommendation

Our findings support the need for urgent efforts to strengthen existing strategies to cope with CRD mortality through reduction in air pollution, planned urbanization, and investment in human capital to achieve Sustainable Development Goals for health. Although CRDs were the third leading cause of death worldwide in 2017, these diseases have received less attention than other non-communicable diseases, such as cardiovascular diseases and neoplasms.

## Data Availability

The datasets used and/or analysed during the current study are available from the corresponding author on reasonable request.

## Abbreviations

CRD: Chronic Respiratory Diseases
GDP: Gross Domestic Product per capita
PM2.5: Particles measuring less than 2.5 μm in aerodynamic diameter
COPD: Chronic obstructive pulmonary disease
CMRCRD: Crude mortality rate from chronic respiratory diseases
GBD: Global Burden of Disease
IHME: Institute for Health Metrics and Evaluation
UNDP: United Nations Development Programme
PPP: Purchasing power parity
UNDESA: United Nations Department of Economic and Social Affairs
AIC: Akaike’s Information Criteria
ML: Maximum likelihood
FGLS: Feasible Generalised Least Square
SD: Standard Deviation
Y-O-Y-: Year-on-year
